# Differential responses in some quinoa genotypes of a consortium of beneficial endophytic bacteria against bacterial leaf spot disease

**DOI:** 10.3389/fmicb.2023.1167250

**Published:** 2023-04-13

**Authors:** Ayman Badran, Nerhan A. Eid, Amr R. Hassan, Henda Mahmoudi

**Affiliations:** ^1^Department of Genetic Resources, Desert Research Center, Cairo, Egypt; ^2^Department of Plant Protection, Desert Research Center, Cairo, Egypt; ^3^Directorate of Programs, International Center for Biosaline Agriculture (ICBA), Dubai, United Arab Emirates

**Keywords:** *Chenopodium quinoa*, endogenous bacteria, *Pseudomonas syringae*, amino acids, PGPB

## Abstract

Many effective plant-microbe interactions lead to biological changes that can stimulate plant growth and production. This study evaluated the effect of the interaction between quinoa (*Chenopodium quinoa* Willd.) and endophytic bacterial strains on differential responses under biotic stress. Four strains of endophytic bacteria were used to inoculate three quinoa genotypes. Endophytic bacteria, isolated from the endosphere of healthy genotypes of quinoa plants, were used to evaluate their biocontrol activity against *Pseudomonas syringae* on quinoa plants, which causes leaf spot disease, depending on some different parameters. Quinoa genotype plants were treated with four treatments: pathogenic bacteria only (T1), internal bacteria only (T2), pathogenic bacteria + endogenous bacteria (T3), and untreated as the control (T4). The results indicated that there was a significant difference between chlorophyll content index of infected plants without bioagent (untreated) compared to plants bio-inoculated with endophytic bacteria. The highest mean disease incidence was on the plants without bacterial inoculum (90, 80, and 100%) for quinoa genotypes G1, G2, and G3, respectively. The results showed that there were significant differences in the weight of grains/plant, as the value ranged from 8.1 to 13.3 g when treated with pathogens (T1) compared to the treatment with pathogens and endogenous bacteria (T3), which ranged from 11.7 to 18.6 g/plant. Decreases in total aromatic amino acids appeared due to the pathogen infection, by 6.3, 22.8, and 24.1% (compared to the control) in G1, G2, and G3, respectively. On the other hand, genotype G3 showed the highest response in the levels of total aromatic and total neutral amino acids. The endophytic strains promoted quinoa seedling growth mainly by improving nutrient efficiency. This improvement could not be explained by their ability to induce the production of amino acids, showing that complex interactions might be associated with enhancement of quinoa seedling performance by endophytic bacteria. The endophytic bacterial strains were able to reduce the severity of bacterial leaf spot disease by 30, 40, and 50% in quinoa genotypes G1, G2, and G3, respectively, recording significant differences compared to the negative control. The results indicated that, G1 genotype was superior in different performance indicators (pathogen tolerance index, yield injury %, superiority measure and relative performance) for grain weight/plant under pathogen infection condition when treated with endophyte bacteria. Based on this study, these bacterial strains can be used as a biotechnology tool in quinoa seedling production and biocontrol to diminish the severity of bacterial leaf spot disease.

## 1. Introduction

Climate change-related land desertification and salinization are increasing at a worrying speed, driving the demand for novel crop cultivation concepts to ensure food security. One prime candidate is the pseudocereala quinoa (*Chenopodium quinoa* Willd.), whose seed has an outstanding nutritional value (Vega-Galvez et al., 2010; Tabatabaei et al., 2022). In 2020, world production of quinoa was 175,188 tons, led by Peru and Bolivia with 97% of the total when combined (FAOSTAT, 2020). Quinoa plants are infected with many bacterial and fungal plant diseases (Li et al., 2017). Fonseca-Guerra et al. (2021) isolated *Pseudomonas* sp. from quinoa leaves; its symptoms appeared in the form of dark brown spots and apical necrosis, thus causing significant damage in crops. The large microbial diversity necessitates the search for different, more efficient bacterial strains to enhance plant capability to colonize quinoa roots (Labanca et al., 2020). Endogenous bacteria decrease the severity of the bacterial pathogen, reduce the rate of disease, and thus diminish the economic loss of the crop.

Bacterial diseases are among the most critical pathogens that may lead to great collapse in the quinoa crop and affect the quantity and quality of the crop. Bacterial leaf spot is remarkable disease of foliage plants as well as vegetables plants. These bacterial diseases may molder leaves, petioles and stems rendering infected plants unsightly and unsalable. The disease is characterized by circular, gray to black, water-soaked lesions on the leaves. Symptoms on leaves included leaf blight and white and brown spots on the leaf surface (Myung et al., 2011). Eid et al. (2019) evaluated leaf spot disease and isolate *pseudomonas* as causative agent from different plants in Egypt.

Bacterial leaf spot disease is important disease of foliage and flowering ornamental plants as well as vegetables plants. This bacterial disease may destroy leaves, petioles and stems rendering infected plants unsightly and unsalable. The disease is characterized by circular, gray to black, water-soaked lesions on the leaves. The lesions coalesce, become irregular in shape, dry and dark brown with age (Burgess et al., 1986). Among them, bacterial spot is the most problematic devastating disease in most of the tomato growing regions around the world (Sharma and Bhattarai, 2019). The incidence of bacterial disease ranged from 48 to 95% on lettuce in Turkey (Ozyilmaz and Benlioglu, 2018). Bacterial leaf spot of onion (*Allium cepa* L.) was observed in fields of Korea with incidence varying from 95 to 100%. Symptoms on leaves included leaf blight and white and brown spots on the leaf surface (Myung et al., 2011).

One of the biggest challenges in quinoa production is maintaining high productivity and minimizing the harmful environmental effects of low fertilization efficiency and long-term use of the soil (Cárdenas-Castillo et al., 2021). The identification of beneficial microorganisms that can improve agricultural production is one of the solutions. In this context, inoculants based on endophytic bacterial strains suggest a sustainable alternative.

Quinoa seedling preparation, one of the main stages of its production, is conducted in nurseries. These conditions can favor beneficial bacterial inoculation *via* micropropagation, which improves crop production at the early stage.

Crop protection is still largely associated with the use of chemical products, despite its negative effects on the environment (Pilet-Nayel et al., 2017). Therefore, the gradual integration of new practices must take into account the environmental and social dimension (Purvis et al., 2019). The continuous growth in productivity and international trade leads to Increased incidence of some diseases, which resulted in the use of more pesticides, which in turn increases environmental pollution (Pilet-Nayel et al., 2017). Therefore, the use of microorganisms is a possible way to reduce pollution and inconveniences associated with the use of synthetic chemicals and reduce their negative impact on the environment clearly (Compant et al., 2005). So, it has become evident the importance of using multiple economic approaches to control plant pathogens while preserving the environment.

Throughout their evolution, plants have developed a complex set of mechanisms for environmental adaptation. One mechanism is association with beneficial microorganisms, such as endophytic and rhizosphere bacteria known as plant growth-promoting bacteria (PGPB). PGPB have been explored as pathogen antagonists and bio-stimulants of plant growth, suggesting an ecofriendly alternative to pesticides and chemical fertilizers in sustainable agriculture (Olanrewaju et al., 2017). The plant-bacteria interaction, through a complex array of mechanisms, can result in plant growth promotion due to increased nutrient uptake, nitrogen fixation, or phytohormone production, or indirectly due to phytopathogen suppression (Berg, 2009). Plant receptors of bacterial signals are known to recognize phytopathogenic bacteria or be involved in the identification of beneficial microorganisms by plants (Carvalho et al., 2016). Likewise, the role of free amino acids and polyamines has been shown to alter during the interaction between plants and microorganisms. Both plant-phytopathogenic bacteria and plant-beneficial microorganism interaction could result in significant changes in polyamine metabolism of the host and/or microbe partners, thus revealing this to be a complex and dynamic process (Terra et al., 2019).

Plant genetic factors might contribute to the increased efficiency of plant-bacteria interaction, causing plant physiological changes that culminate in the modulation of plant growth and development (Carvalho et al., 2016). The endophytic bacterial strains with different characteristics differently alter the plant response regarding increasing amino acid content. To test this hypothesis, we designed an experiment to evaluate whether quinoa seedlings inoculated with endophytic bacteria could induce pathogen resistance reactions and reduce the need for chemical fertilization. Increasing environmental concerns and the search for a more sustainable agriculture have led research to intensify the development of biofertilizers. However, the correct and efficient use of microorganisms as inoculants requires more knowledge about their benefits and impacts. Thus, the objective of this study was to evaluate the effect of consortium of bacterial inoculants on the development of quinoa plants and resistance to bacterial leaf spot disease using the physiological and biochemical aspects of plant-bacteria interaction.

## 2. Materials and methods

### 2.1. Tested genotypes

Three genotypes of quinoa were selected among a large group of genetic material provided by the International Center for Biosaline Agriculture (ICBA): genotype G1 (CO-KA-1873), genotype G2 (CO-KA-2300), and genotype G3 (CO-KA-1901).

### 2.2. Isolation of endophytic bacterial strains

Four endophytic bacterial strains were isolated from the endosphere of healthy genotypes of quinoa plants, QF3 and QF4 were isolated from quinoa genotype (CO-KA 1982), QC5 was isolated from quinoa genotype 132 (D 12123) and QD4 was isolated from quinoa genotype 64 (CO-KA 1830). The age of the plants used for isolation was 60 days. For isolation of endophytic bacteria, the quinoa roots, stems and leaves (1 cm pieces) were washed by running tap water with two drops of tween 20 (as wetting agent) then surface sterilized by ethanol 70% for 2 min. Followed by sodium hypochlorite 2% for 1 min. After that, the samples were washed in sterile distilled water for 3 times, drying on sterilized filter paper under aseptic conditions (laminar air flow). The last washing distilled water was plated onto nutrient agar medium for 48 h, incubated at 28 ± 2C° to confirm that the surface of plant pieces was efficacious purified the parts of sterilized roots, stems, and leaves were macerated in 5 ml of 12.5 mM potassium phosphate buffer (pH 7.0) with sterile mortar and pestle. Tissue extracts were then sequent diluted in potassium phosphate buffer (pH 7.0) and plated on tryptic soy agar (TSA) plates. Incubation was carried out at 28°C for 1–7 days to allow growth of endophytic bacteria. The plates were incubated at 28 ± 2°C for 1–7 days or pending growth was observed (Das et al., 2007).

### 2.3. Characterization of endophytic bacterial strains

Four endophytic bacterial strains were isolated from the endosphere of healthy genotypes of quinoa plants and characterized by their antagonistic activities against *Pseudomonas syringeae*, bacterial strains incubated at 28–30°C for 4 days. To determine production of indole-3-acetic acid (IAA) according to Bric et al. (1991), Cell free culture filtrate was gained by centrifuging 10 ml culture at 10000 rpm for 20 min at 4°C., The amount of gibberellic acid (GA3) in the ethyl acetate phase was determined by the UV spectrophotometer at 254 nm versus control blank (Mitter et al., 2002), % antioxidant (Burits and Bucar, 2000), total phenols (Kumar and Min, 2011), hydrogen cyanide production, the filter padding in each tube was soaked with 2 ml of sterile picric acid solution (Picric acid 2.5 g/l + Na_2_Co_3_ 12.5 g/l) under aseptic condition and the lids were closed. The tubes were sealed with parafilm in order to contain gaseous metabolites produced by the antagonists and to allow for chemical reaction with picric acid present in the filter paper padding. Wei et al. (1991), and siderophore production according to Alexander and Zuberer (1991), standard solution of deferoxamin mesylate (DFOM) with concentrations (15,30,45,60,90,105,120 and 135 mM) was prepared to calibrate the assay of the collected samples. A standard curve for the modified Chrome Azurol S (CAS) assay was prepared by analyzing the absorbance (630 nm) of each standard solution. The concentration of siderophores in the culture filtrate was measured by modified CAS assay method.

### 2.4. Evaluation of the antagonistic interaction among selected isolates of endophytic bacterial strains

The inhibitory effect of the selected isolates was measured based on the formation of an inhibition zone around the organism on King’s medium agar plate. The test was carried out among selected strains according to Berga et al. (2001).

### 2.5. Identification of endophytic bacterial strains

The BLAST database of the National Center for Biotechnology Information was used to compare the resolved sequences of the most efficient bacterial isolates with known 16S rDNA sequences (Altschul et al., 1997). Determination of phylogenetic relationships was analyzed by the program Phylogenetic Analysis CLC Genomics Workbench version 4.5.1 (Vinnere et al., 2002).

The most efficient antagonistic isolates were identified by 16S rRNA sequence. Isolation of cellular DNA was performed as described by Ausubell et al. (1987) and amplification of 16S rDNA according to Lane et al. (1985) using the universal 16S primers [F1 5′ AGAGTTT(G/C) ATCCTGGCTCAG 3′ R1 5’ ACG(G/C) TACCTTGTTACGACTT 3′]. PCR was run on a Gene Amp PCR System 2400 thermal cycler (Perkin Elmer) and then DNA was amplified according to Lane et al. (1985). The resulting PCR product sizes ranged from 1450 to 1500 bp. The PCR products were purified using QIA Quick PCR Purification Kit (Qiagen). The sequencing was performed in two directions using the previously described primers (Lane et al., 1985) in GATC Company (Germany). Sequencing data were analyzed by two different computer alignment programs, DNAStar (DNASTAR, Inc., United States) and Sequence Navigator (Perkin Corp., United States).

### 2.6. Evaluation of endophytic bacteria against bacterial leaf spot of quinoa *in vivo*

A greenhouse trial was conducted during 2021 in Al-Ismailia Governorate, Egypt, using mixed culture of the most efficient endophytic bacterial strains: *Pseudomonas taiwanensis* QF3, *Bacillus velezensis* QC5, *Bacillus subtilis* QF4, and *Pseudomonas putida* QD4.

The response of the three quinoa genotypes to the previous four treatments was evaluated in a randomized complete block design using three replicates.

#### 2.6.1. Inoculum preparation of antagonistic bacteria

The antagonistic bacteria were inoculated in nutrient broth medium (individually) and inoculated at 28 ± 2°C for 2 days. After the incubation period, the bacterial cultures were mixed. The cell density of the mixed culture was ~10^9^ cfu/ml to use as a standard inoculum.

#### 2.6.2. Inoculum preparation of pathogenic bacteria

The 48-h-old culture of *Pseudomonas syringae* was inoculated in nutrient broth medium individually for 48 h at 28 ± 2°C. The optical density (OD) was adjusted at ~10^6^ cfu/mL for the inoculum to be used as a standard inoculum.

#### 2.6.3. Inoculation treatments

Our study was conducted to investigate the effect of antagonistic endophytic bacterial strains on the severity of bacterial leaf spot disease. The seeds were soaked in the mixed culture of antagonistic bacteria and then cultivated in sapling trays. After 2 weeks, the seedlings were transferred to pots and the inoculation treatments were applied as follows:

Quinoa genotype plants treated using pathogenic bacteria only (T1), plants treated using endogenous bacteria only (T2), plants treated using pathogenic bacteria + endogenous bacteria (T3), and sterile media was used in untreated plants. Untreated quinoa plants as the control (T4).

#### 2.6.4. Antagonistic bacterial treatments

Five mL of mixed liquid culture consisting of 5 g peptone, 3 g beef extract, and 10 g glucose of four isolated endophytic bacteria were added to the pots by supplementing to the soil in a 2-cm hole beside the seedlings after transplanting.

#### 2.6.5. Pathogenic bacterial treatment

The pathogenic bacterium *Pseudomonas syringae* was used as foliar application on the seedlings after 48 h of antagonistic bacterial treatment.

Re-inoculation of the antagonistic bacteria was carried out for quinoa plants (1 month after transplanting). Incidences of leaf spot disease were recorded after 10 days of infection according to Li et al. (2008).

#### 2.6.6. Compatibility test between the selected antagonists *in vitro*

Cross interaction between selected bacterial isolates (QF3, QC5, QF4, and QD4) was examined for each other to know whether any antibiosis was presented among them. We concluded that no antibiosis was noticed for any combination of tested isolates.

### 2.7. Growth parameters

#### 2.7.1. Vegetative growth and agronomic characteristics

Sixty days after planting, vegetative growth parameters such as plant height (cm), fresh and dry weight of shoot (g/plant), leaf area (cm^2^), and chlorophyll content index were estimated, three CCI readings per leaf, including one reading around the midpoint of leaf blade and two readings 3 cm apart from midpoint. Chlorophyll was measured using chlorophyll Meter SPAD-502 (MINOLTA C., LTD JAPAN 78923067). After harvesting, grain yield/plant and 100-grain weight (g) were estimated.

#### 2.7.2. Determination of disease incidence

The disease incidence percentage was calculated by using the following formula:

Disease incidence (%) = (Number of diseased leaves in treatment/ Total number of leaves/treatment) × 100.

#### 2.7.3. Determination of amino acids

The determination of free amino acids was performed by ultra-performance liquid chromatography mass spectrometry (UPLC-MS). Samples of young leaves were collected, macerated in liquid nitrogen, and lyophilized. Extraction was performed from 100 mg of lyophilized material, homogenized with 1 ml of methanol water (80,20, v:v) in an ultrasonic bath at 30°C for 15 min, centrifuged at 10,000 rpm for 5 min, and the supernatant collected for analysis. The extracted samples were analyzed directly on an Acquity UPLC-MS (QTOF, Micromass-Waters, Manchester, United Kingdom). The chromatographic separation was done in a Waters Acquity C18 BEH analytical column (150 mm × 2.1 mm i.d, 1.7 μm) according to Tezotto et al. (2016). Output data were obtained in the range of 50 at 300 m/z. For quantification of amino acids, calibration curves were produced by the injection of a known concentration of the standards. In our investigation free amino acids in leaves were quantified to monitor the changes due to plant-pathogen interaction. Such infection, with prolonged exposure, may result in changes in amino acids profile in the seeds, particularly in terms of storage protein quantity. However, seed protein, and amino acids profile were not examined in our project.

#### 2.7.4. Tolerance indices of tested genotypes against pathogen

Tolerance Indices of tested genotypes against pathogen were calculated as follow:

1. Pathogen tolerance index (PTI): PTI = (Yp) × (Yd)/(Ýp)2 according to Fernandez (1992).
2. Yield injury % (YI): YI = (Yp-Yd)/Yp × 100 according to Blum et al. (1983).
3. Superiority measure (SM): SM = Yd/Yp according to Lin and Binns (1988).
4. Relative performance (RP): RP = (Yd/Yp)/R according to Abo-Elwafa and Bakheit (1999).

Where, Yp = grain weight/plant using pathogen & endophytic bacteria treatment (T3); Yd = grain weight/plant using pathogen (T1); Ýp = Mean grain weight/plant of all genotypes using pathogen & endophytic bacteria treatment (T3); Ýd = Mean grain weight/plant of all using pathogen; R = (Ýd/Ýp).

### 2.8. Statistical analysis

Statistical significance was calculated using the analysis of variance procedure in SAS program (version 9.0). Differences between the means of tested genotypes in treatments were separated by the least significant difference (LSD) test at the 0.05 *P* level.

## 3. Results and discussion

### 3.1. Identification of antagonistic bacteria by molecular analysis

**Figure 1:**
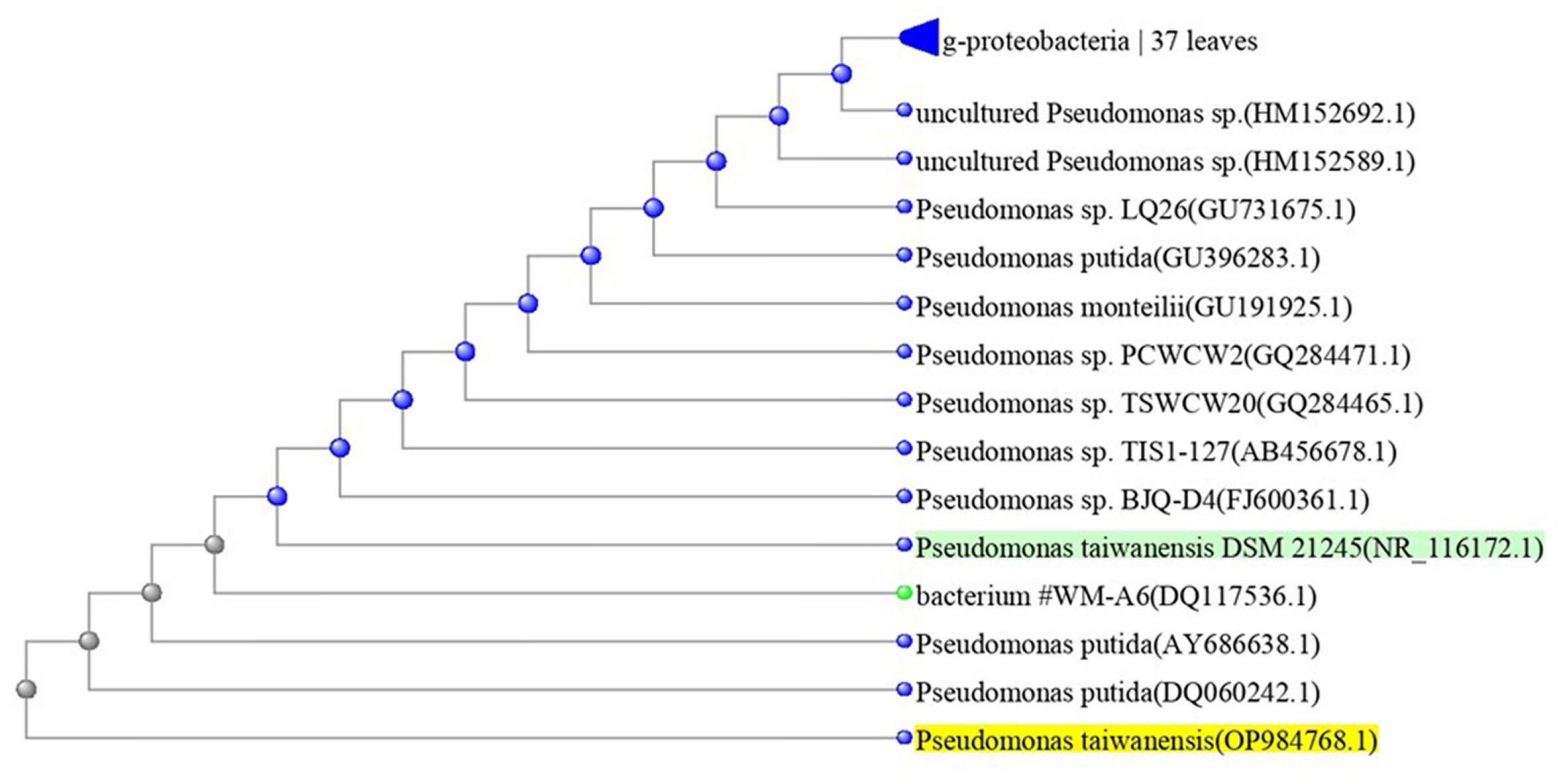
Overview of phylogenetic tree, based on 16S rRNA gene sequences, showing the relationships between isolate QF3 and related taxa, which had high identity with *Pseudomonas taiwanensis* strain Pst1 (OP984768). Unrooted phylogenetic tree of isolate QF3 rDNA seq. This topology was obtained using all known complete or almost complete sequences from *Pseudomonas* strains and the percentage values indicating the results of a bootstrap analysis (500 replications, values above 50%) and maximum-parsimony analyzes are shown by *p* < 0.01.

**Figure 2:**
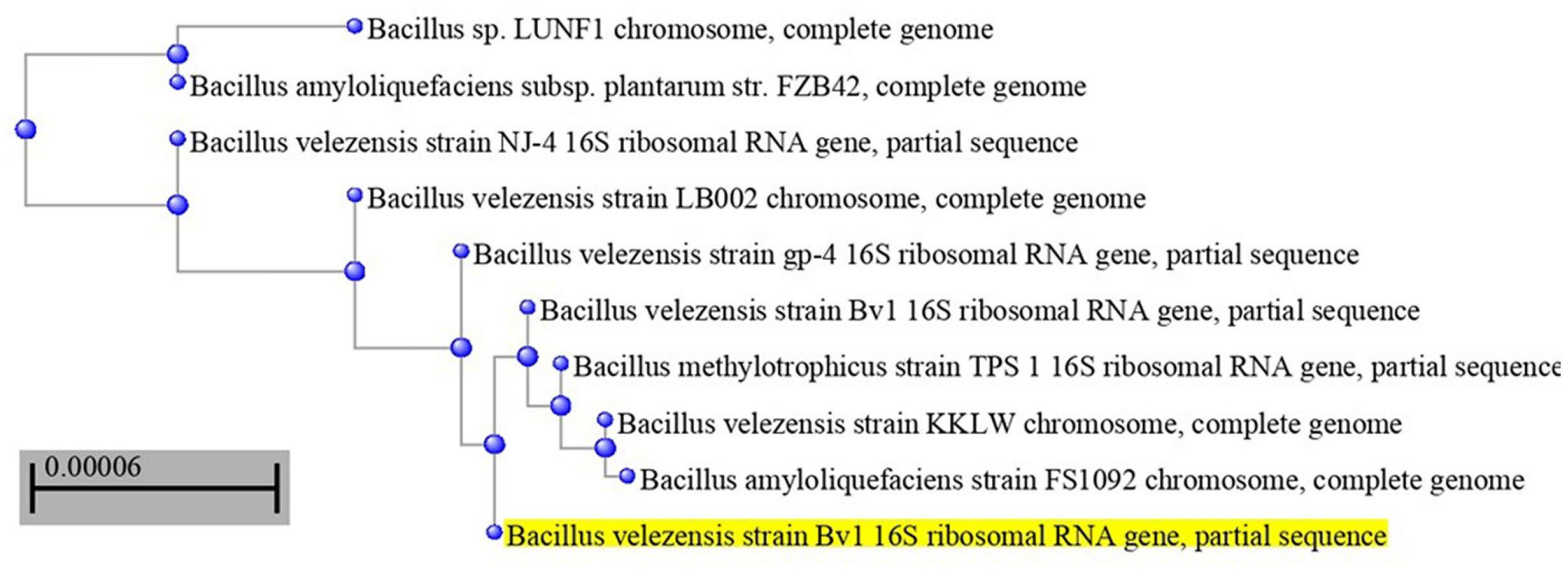
Overview of phylogenetic tree, based on 16S rRNA gene sequences, showing the relationships between isolate QC5 and related taxa, which had high identity with *Bacillus velezensis* strain Bv1 (OP984765). The tree was reconstructed from the core genomes of type strains of species from the *Bacillus* group (350 genes). Bootstrap values >50%, based on 1,500 replicates, are indicated on branch points.

**Figure 3:**
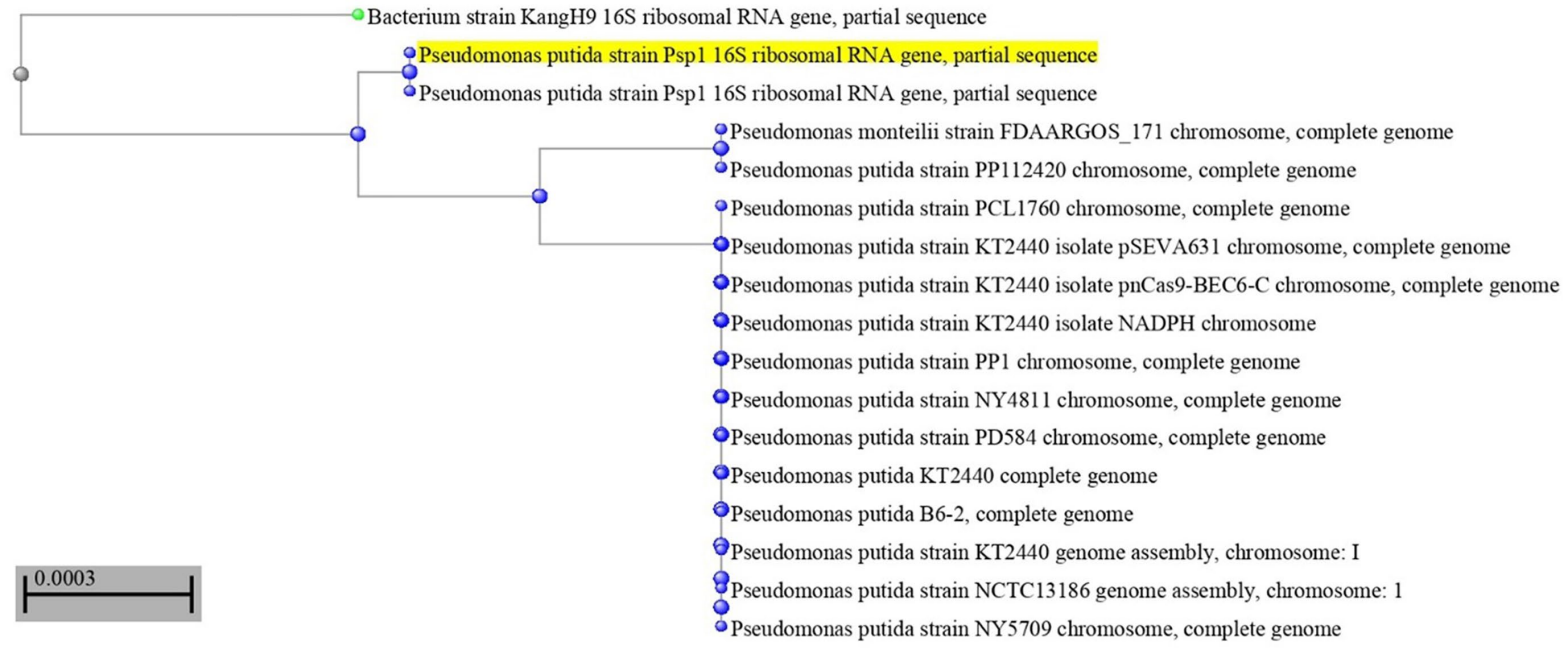
Overview of phylogenetic tree, based on 16S rRNA gene sequences, showing the relationships between isolate QD4 and related taxa, which had high identity with *Pseudomonas putida* strain Psp1 (OP984769). The percentage of trees in which the associated taxa clustered in the bootstrap test (1,000 replicates) is shown next to the branches.

**Figure 4:**
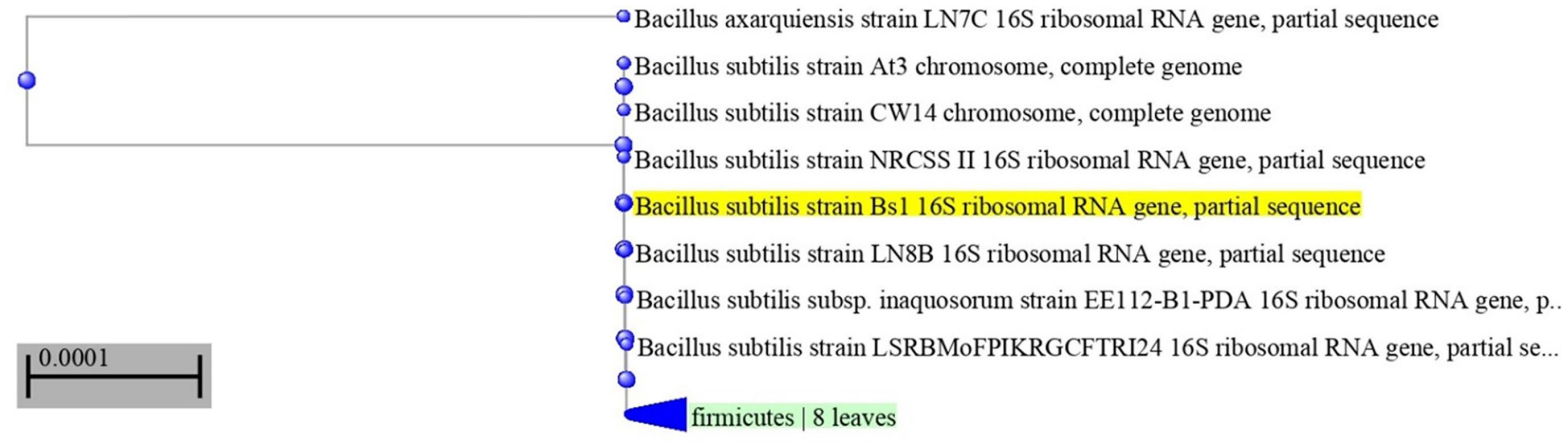
Neighbor-joining phylogenetic tree, based on 16S rRNA gene sequences, showing the relationships between isolate QF4 and related taxa, which had high identity with *Bacillus subtilis* strain Bs1 (OP984766). Phylogenetic tree of 16S rRNA sequences from endophytic bacterium strain QF4 compared with representative members of *Bacillus* genus with more than 98% identity.

The most potent antagonistic bacteria were identified by amplifying and sequencing the 16S rDNA using techniques of Sigma Scientific Services. The results showed that the 16S rDNA sequence of QF3, QC5, QD4, and QF4 isolates had 99% identity with *Pseudomonas taiwanensis* strain Pst1 (OP984768), *Bacillus velezensis* strain Bv1 (OP984765), *Pseudomonas putida* strain Psp1 (OP984769), and *Bacillus subtilis* strain Bs1) OP984766), respectively. Construction of a phylogenetic tree based on comparative analysis of the 16S rRNA genes was performed with the use of various algorithms implemented in CLC Genomics Workbench version 4.5.1. The phylogenetic analysis based on 16S rRNA gene sequences indicated that strains QF3, QC5, QD4, and QF4 formed a phyletic lineage within *Pseudomonas taiwanensis* strain Pst1 (OP984768), *Bacillus velezensis* strain Bv1 (OP984765), *Pseudomonas putida* strain Psp1 (OP984769), and *Bacillus subtilis* strain Bs1)OP984766), respectively (Figures 1–4).

### 3.2. Performance of endophytic bacteria upon different genotypes of quinoa

#### 3.2.1. Vegetative and agronomic characteristics

The effects of plant growth-promoting endophytic bacteria (PGPEB) as bioagents in quinoa plants in the presence of phytopathogenic bacteria are presented in Tables 1–4.

**Table 1:** Effects of endophytic bacteria on root length, shoot length, and leaf area of the different quinoa genotypes.

Treatment	Shoot length (cm)			Root length (cm)			Leaf area (cm^2^)		
	G1	G2	G3	G1	G2	G3	G1	G2	G3
Pathogen	20.3	24.0	22.0	11.03	11.16	10.66	10	9	10
Endophytic bacteria	39.3	42.0	39.6	16.26	16.23	15.20	19	19	21
Pathogen and endophytic bacteria	30.3	28.6	28.3	13.90	14.10	14.03	17	17	19
Control	28.0	29.0	27.3	11.73	12.30	12.30	14	15	14
LSD _(0.05)_	1.82			4.47			1.46		

LSD = least significant difference.

**Table 2:** Effects of endophytic bacteria on shoot fresh and dry weight of the different genotypes of quinoa plants.

Treatment	Shoot fresh weight (g)			Shoot dry weight (g)		
	G1	G2	G3	G1	G2	G3
Pathogen	5.5	5.2	6.1	0.6	0.8	1.0
Endophytic bacteria	11.6	10.0	10.2	1.4	1.6	1.3
Pathogen and endophytic bacteria	8.5	7.9	8.2	1.2	1.2	1.2
Control	7.9	7.9	7.3	1.0	1.0	1.1
LSD _(0.05)_		1.01			0.65	

LSD = least significant difference.

**Table 3:** Effects of endophytic bacteria on root fresh and dry weight of the different genotypes of quinoa plants.

Treatment	Root fresh weight (g)			Root dry weight (g)		
	G1	G2	G3	G1	G2	G3
Pathogen	0.3	0.6	0.6	0.1	0.1	0.1
Endophytic bacteria	1.1	1.2	1.0	0.9	1.1	0.9
Pathogen and endophytic bacteria	0.6	0.9	0.8	0.5	0.6	0.5
Control	0.8	0.7	0.8	0.4	0.4	0.4
LSD _(0.05)_	0.23				0.02	

LSD = least significant difference.

**Table 4:** Effects of endophytic bacteria on 1,000-grain weight and grain weight/plant of some different quinoa genotypes.

Treatment	1,000-grain weight (g)			Grain weight/plant (g)		
	G1	G2	G3	G1	G2	G3
Pathogen	1.00	1.11	1.01	13.3	8.1	11.2
Endophytic bacteria	2.90	3.10	3.00	19.0	13.0	18.3
Pathogen and endophytic bacteria	1.90	1.70	1.70	18.6	11.7	17.4
Control	2.10	1.49	2.01	15.1	10.3	14.3
LSD _(0.05)_	0.122			0.670		

LSD = least significant difference.

The plants that were inoculated with bioagents showed a positive effect on growth parameters. The data indicated that the inoculation treatments with biocontrol agents had the highest significant plant growth in the presence of pathogenic bacteria compared with the control treatments. Quinoa plant bacterization with endophytic bacteria registered a highly significant increase in shoot and root length (cm), fresh and dry weight of shoot (g/plant), fresh and dry weight of root (g/plant), leaf area (cm), and 1,000-grain weight (g). This means that the biocontrol agents suppress the activity of *Pseudomonas syringae* as well as stimulate the growth of quinoa plants.

Infected plants without bioagent showed a significant reduction in plant growth traits (i.e., shoot and root length (cm), fresh and dry weight of shoot (g/plant), fresh and dry weight of root (g/plant), leaf area (cm), and 100-grain weight (g)). The highest significant increase in growth traits was observed with the endophytic bacterial treatment. The increment in previously mentioned attributes for endophytic bacteria-treated plants was particularly significant in most cases vis-à-vis all other treatments. Endophytic bacteria play an essential role in improving crop growth during biotic stress conditions. The endophytic bacterial treatment increased shoot and root length (cm), fresh and dry weight of shoot (g/plant), fresh and dry weight of root (g/plant), leaf area (cm^2^), and 1,000-grain weight (g) of quinoa plants compared with un-inoculated plants in the presence of phytopathogenic bacteria. The fresh weight of roots was determined to give 1.1, 1.2, and 1.0 g/plant for quinoa genotypes G1, G2, and G3, respectively, with endophytic bacterial inoculum, against 0.3, 0.6, and 0.6 g/plant for quinoa genotypes G1, G2, and G3, respectively, with un-inoculated plants in the presence of phytopathogenic bacteria. Similar results were observed by Mahdi et al. (2020), who found that *Bacillus licheniformis* QA1 and *Enterobacter asburiae* QF11 treatment significantly increased the fresh and dry yield and plant height of quinoa plants.

#### 3.2.2. Photosynthetic pigments

**Figure 5:**
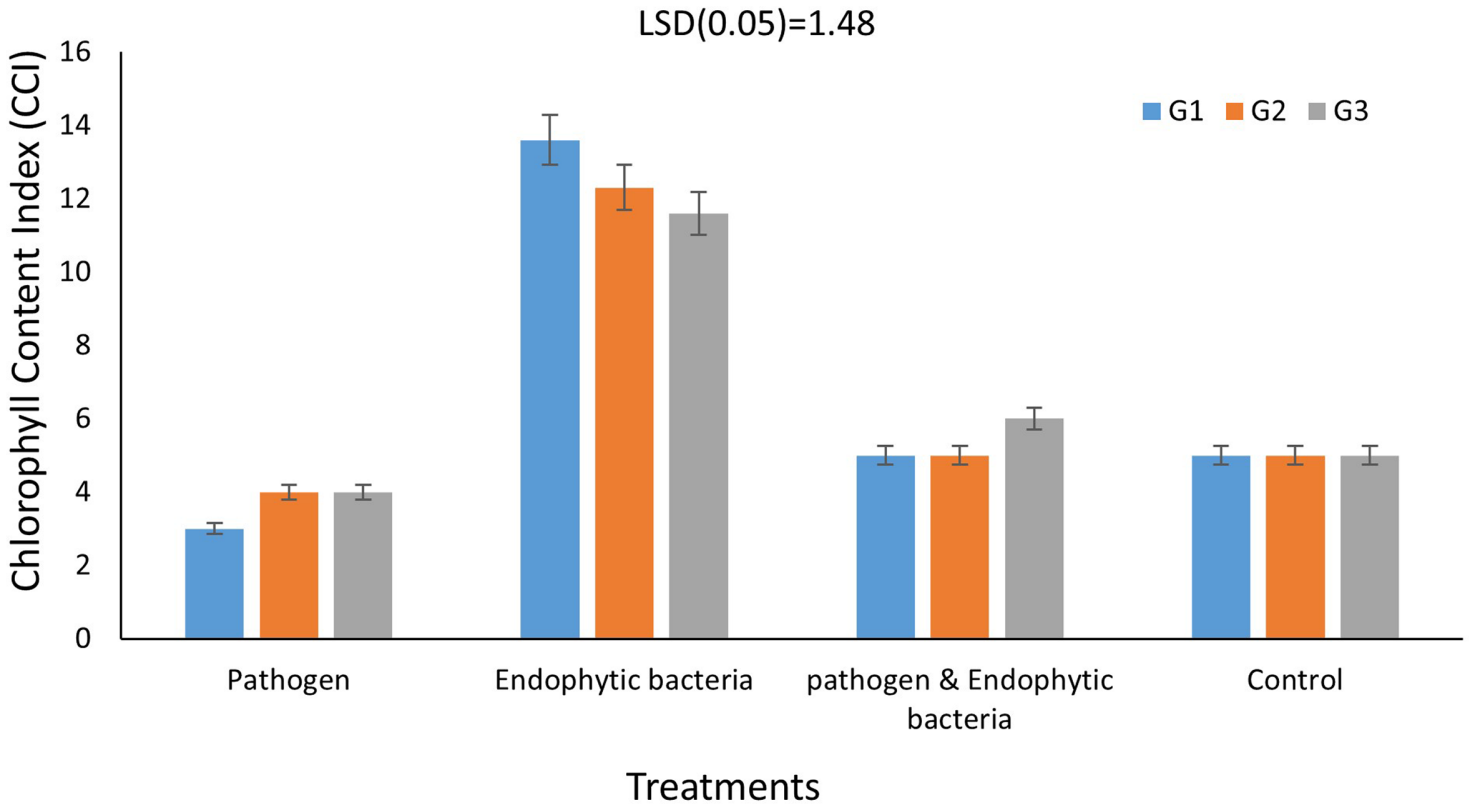
Effects of endophytic bacteria on chlorophyll content index of different genotypes of quinoa (G1, G2, G3). Data are means of three replicates. Differences between the means of tested genotypes under treatments were differentiated by least significant difference (LSD) test at the 0.05 *P* level.

Chlorophylls are considered the main photosynthetic pigments that harvest light energy used to start photosynthesis processes. Such processes are the main motive force for plant growth and development. The total chlorophyll content index (CCI) of fresh leaves of quinoa plants is shown in Figure 5 for each genotype. The CCI of infected plants without bioagent (untreated) was significantly less than that of plants bio-inoculated with endophytic bacteria. Decreases in photosynthetic pigments might be due to the destruction of chlorophyll by increased activity of chlorophyll-degrading enzymes, which results in leaf necrotic areas under stress conditions. The highest significant values of CCI were recoded with endophytic bacterial inoculum treatment. At the cellular level, the bacterial infection causes oxidative damage resulting from higher levels of reactive oxygen species (ROS), which negatively affect photosystem integrity and decrease chlorophyll content, thus affecting the development and productivity of the plant (Osdaghi et al., 2021). A similar result was reported by Mahdi et al. (2020), who observed that inoculation with *Bacillus licheniformis* QA1 significantly increased the leaf CCI in quinoa plants.

### 3.3. Leaf spot disease expression

**Figure 6:**
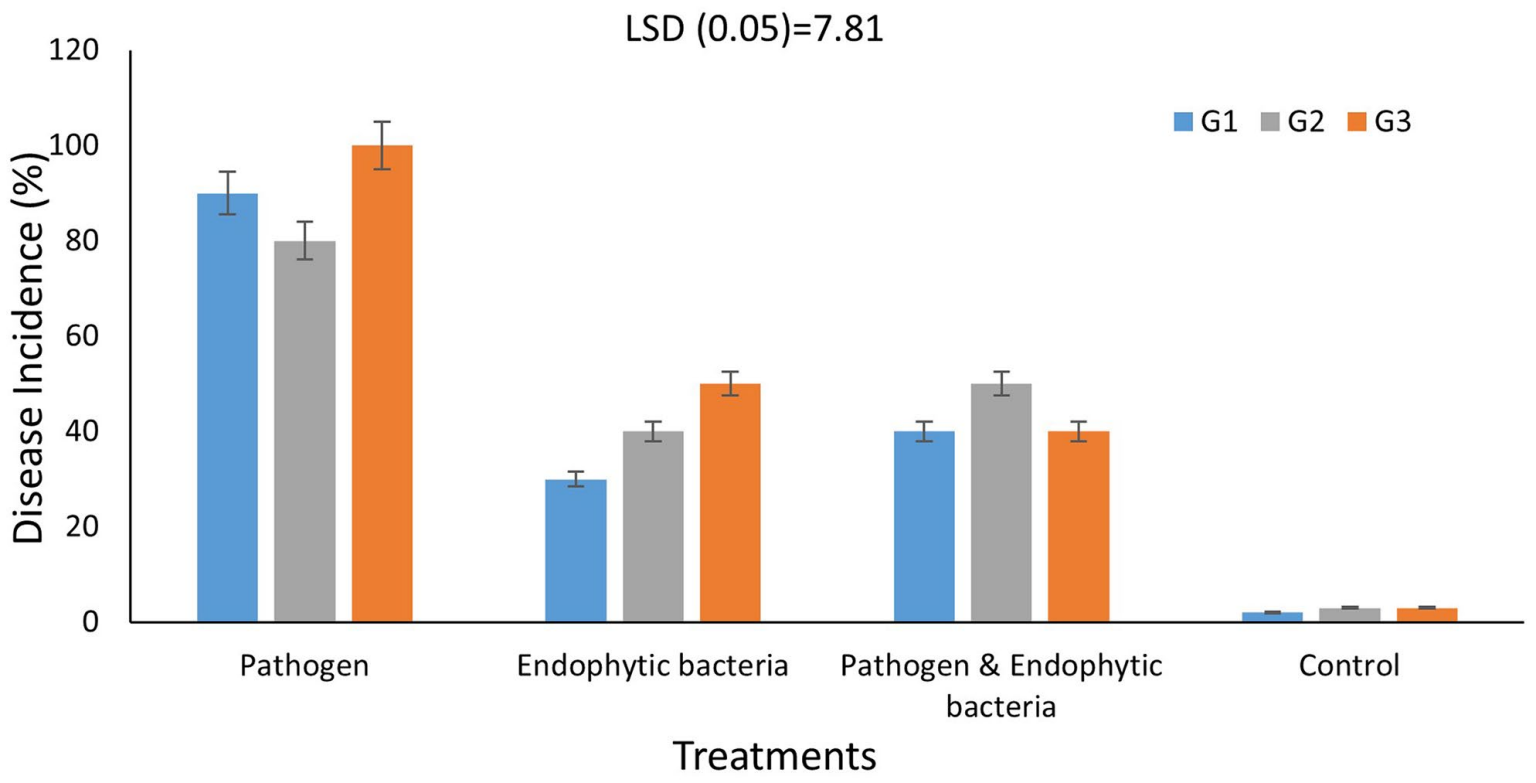
Effects of endophytic bacteria on disease incidence (%) of some different genotypes of quinoa plants (G1, G2, G3). Values of disease incidence (as a percentage) data were transformed by arcsine before analysis of variance. Data are means of three replicates. Differences between the means of tested genotypes under treatments were differentiated by least significant difference (LSD) test at the 0.05 *P* level.

Disease incidence in different genotypes of quinoa plants with different treatments is presented in Figure 6. The typical symptoms of leaf spot were developed on the leaves 10 days after inoculation. The averages of disease incidence with endophytic bacteria as bioagent were 30, 40, and 50% for quinoa genotypes G1, G2, and G3, respectively, and there was a significant difference compared with the control treatment (five plants as replicates). The highest mean disease incidence was on the plants without bacterial inoculum (90, 80, and 100%) for quinoa genotypes G1, G2, and G3, respectively, whereas the results indicated that the mixture of bacteria improved the induced plant resistance against the pathogenic bacteria. Backer et al. (2018) reported similar results. Some bacteria support plant growth indirectly by improving growth-restricting conditions either *via* the production of antagonistic substances or by inducing host resistance toward plant pathogens. Since associative interactions of plant and microorganisms must have come into existence because of convolution, the use of bioinoculants forms one of the vital components for a long-term sustainable agricultural system (Backer et al., 2018). Le et al. (2020) stated that the application of *Paenibacillus elgii* JCK-5075 caused effective suppression of the development of red pepper bacterial leaf spot in pot experiments with control values of 67%. According to Lalitha (2017), various plant growth-promoting rhizobacteria bring about induced systemic resistance (ISR) and therefore provide resistance against plant pathogens. ISR was reported to be associated with many defense enzymes, including ascorbate peroxidase (APX), β1,3-glucanase, catalase (CAT), chitins, lipoxygenase (LOX), peroxidase (PO), phenylalanine ammonia lyase (PAL), polyphenol oxidase (PPO), proteinase inhibitors, and superoxide dismutase (SOD).

Our study recognized the endophytic bacteria *Pseudomonas taiwanensis* QF3, *Bacillus velezensis* QC5, *Bacillus subtilis* QF4, and *Pseudomonas putida* QD4 as suitable inoculants for quinoa cultivation with potential for alleviating biotic stress in quinoa plantlets. These bacteria possess desirable characteristics of PGPB, such as antagonistic activities against phytopathogens, siderophores, auxins, and cyanide production.

These obtained results are in harmony with those obtained by many investigators such as Cipriano et al. (2021), who confirmed that using endophytic bacteria promoted sugarcane seedling growth mainly by improving nutrient efficiency. Previously, numerous reports studied the endophytic bacterial community isolated from leaves, stems, and roots of quinoa plants, including *Paenibacillus* sp., *B. megaterium*, and *Pseudomonas* sp., which has been previously characterized as a quinoa leaf endophyte (Ortuño et al., 2013; Zahoor et al., 2022).

#### 3.3.1. Amino acid production

The free amino acid (FAA) profiles in the leaves of the quinoa genotypes under study as affected by the pathogen and the endophytic bacteria (individually or in combination) are shown in Table 5 compared to the control. The pathogen negatively affects the FAA content (compared to the uninfected plants) among all the quinoa genotypes. The total FAA decreased in the pathogen-treated plants by 5.1, 6.2, and 6.8% in the leaves of G1, G2, and G3, respectively, compared to the control plants. In this context, total acidic amino acids exhibited negligible changes due to the pathogen (compared to the control) in all the studied genotypes. Notable decreases occurred in the total basic and total neutral amino acid levels. Moreover, dramatic decreases in total aromatic amino acids appeared due to the pathogen infection, by 6.3, 22.8, and 24.1% (compared to the control) in G1, G2, and G3, respectively. In this context, the levels of phenylalanine of the infected plants showed the most remarkable decreases: by 7.5% in G1, by 23.8% in G2, and by 26.8% in G3 (compared to the uninfected ones). Phenylalanine, tyrosine, and phenolics are biosynthesized through the shikimate pathway. The shikimate pathway, in plants, is localized in the chloroplast. These aromatic molecules have roles as pigments, antioxidants, signaling agents, electron transport, communication, and the structural element lignan, and as a defense mechanism (Santos-Sánchez et al., 2019). Inhibition of one or more of the enzymes controlling this pathway by pathogen interactions might be a mechanism of pathogenicity.

**Table 5:** Effects of endophytic bacteria on amino acid content of the different genotypes of quinoa seeds.

Type		G1*				G2*				G3*			
		Amino acid weight (g/100 g)											
		T1*	T2*	T3*	T4*	T1*	T2*	T3*	T4*	T1*	T2*	T3*	T4*
Acidic	Aspartic acid	9.7	10.2	9.5	9.3	8.9	10.0	9.3	9.1	9.5	10.3	9.4	9.3
	Glutamic acid	13.0	13.5	13.4	13.2	12.9	13.6	13.3	13.1	13.2	13.7	13.5	13.4
Aromatic	Phenylalanine	3.7	4.8	4.5	4.0	3.2	5.0	4.9	4.2	3.0	5.4	4.8	4.1
	Tryptophan	0.9	1.2	1.0	1.0	0.7	1.1	0.9	0.8	1.0	1.3	1.2	1.1
	Tyrosine	2.9	3.9	3.2	3.0	2.2	3.9	3.1	2.9	2.6	4.3	3.2	3.5
Basic	Arginine	7.8	8.3	8.1	8.0	7.8	8.4	8.2	8.1	7.5	8.4	8.0	7.8
	Histidine	3.1	4.2	3.8	3.9	3.0	4.0	3.6	3.8	3.4	4.4	3.9	3.4
	Lysine	5.0	6.7	6.0	5.5	4.8	6.3	6.1	5.2	5.2	7.0	6.7	5.7
Neutral	Alanine	7.0	7.5	7.3	7.2	6.9	7.4	7.2	7.0	7.0	7.4	7.1	7.1
	Cysteine	2.1	3.0	2.5	2.7	2.1	3.2	2.4	2.9	2.0	3.8	2.7	3.0
	Glycine	10.5	11.3	11.0	10.7	10.3	11.6	11.3	11.0	10.7	11.4	11.1	11.0
	Isoleucine	3.2	4.8	4.1	3.8	3.3	5.0	4.2	3.9	3.1	5.2	4.8	3.5
	Leucine	5.1	6.9	6.1	5.8	5.3	7.0	6.4	5.5	5.0	7.1	6.7	5.8
	Methionine	1.7	2.9	2.2	2.0	1.9	2.3	2.0	1.9	1.7	2.9	2.2	1.5
	Proline	4.0	4.6	4.2	4.1	4.1	4.8	4.4	4.3	4.2	4.6	4.4	4.5
	Serine	6.0	6.5	6.3	6.1	6.1	6.7	6.4	6.2	6.0	6.4	6.2	6.3
	Threonine	3.0	4.2	3.7	3.2	3.5	4.8	3.1	3.0	3.1	5.2	3.0	3.0
	Valine	4.0	5.1	4.6	4.2	3.9	5.8	4.2	4.0	3.4	6.7	4.7	4.3
Total acidic AA		22.7	23.7	22.9	22.5	21.8	23.6	22.6	22.2	22.7	24.0	22.9	22.7
Total aromatic AA		7.5	9.9	8.7	8.0	6.1	10.0	8.9	7.9	6.6	11.0	9.2	8.7
Total basic AA		15.9	19.2	17.9	17.4	15.6	18.7	17.9	17.1	16.1	19.8	18.6	16.9
Total neutral AA		46.6	56.8	52.0	49.8	47.4	58.6	51.6	49.7	46.2	60.7	52.9	50.0

T1*: pathogen, T2*: endophytic bacteria, T3*: pathogen and endophytic bacteria, T4*: control.

G1*: genotype (CO-KA-1873), G2*: genotype (CO-KA-2300), G3*: genotype (CO-KA-1901).

However, inoculation with the endophytic bacteria resulted in consistent increases in leaf FAA content of the three quinoa genotypes compared to the control. Dual inoculation (pathogen and endophytic bacteria) not only counteracted the negative effects of the pathogen infection in terms of leaf FAA, but also by over the healthy untreated plants. In terms of amino acid fractions, acidic, basic, neutral, and especially aromatic amino acid levels increased in response to the dual inoculation treatment for all the genotypes. Noteworthy was that genotype G3 showed a higher response to the dual inoculation as its levels of neutral, basic, and aromatic amino acids increased by 21.4, 17.2, and 26.4%, respectively, compared to the untreated plants. Within the aromatic amino acid group, the prominent increases were related to the levels of tyrosine (22.9–34.5% increase) and phenylalanine (19.0–31.7% increase) vis-à-vis the control. Interestingly, specific amino acids exhibited marked increments due to dual inoculation: lysine, methionine, threonine, leucine, and valine. In general, the superiority of genotype G3 was observed in its response to the pathogen and endophytic bacteria (T3) against the pathogen (T1), and this is evident by the increase in the proportions of most essential amino acids.

Quinoa is a strategic crop because of its high N content and its adaptability to adverse conditions, as nitrogen is one of the elements that plants need in large quantities. Quinoa responds positively to fertilization by nitrogen (Badran et al., 2020; Cárdenas-Castillo et al., 2021). Quinoa is a strategic partner crop for food security as a plant-based protein source (Alandia et al., 2020) and its adaptability to unfavorable growing conditions (Rodriguez et al., 2020; Badran, 2022). As a result of previous research, based on the plant’s response to nitrogen fertilization, bacterial treatments were used that effectively provided nitrogen for quinoa plants through their effect on the synthesis of amino acids within the plant tissues after bacterial treatment.

Our results are in harmony with Almuhayawi et al. (2021), who showed that endophytic bacterium inoculation can increase amino acid levels of the sprouts of three *Chenopodium* species: *C. ambrosoides*, *C. ficifolium*, and *C. botrys*, Endo 2 (strain JSA11).

#### 3.3.2. Tolerance indices of tested genotypes against pathogen

Tolerance indices were calculated to evaluate the tested genotypes of quinoa against Pseudomonas syringae. Weight/plant (g) of the three genotypes under treated with pathogens (T1) compared to the treatment with pathogens and endogenous bacteria (T3) were measured to assess the tolerance indices of the tested genotypes (Table 6). Pathogen tolerance index (PTI) data indicates that the genotype G1 was the most tolerant according to the grain weight of the plant (0.98), while the G2 genotype was the least tolerant to infection with the pathogen (0.37) during the study.

**Table 6:** Tolerance indices of tested quinoa genotypes under the condition of pathogen (T1) and pathogen and endophytic bacteria (T3) for grain weight/plant.

Genotype	Yp	Yd	PTI	YI (%)	SM	RP
G 1	18.60	13.30	0.98	28.49	0.72	1.05
G 2	11.70	8.10	0.37	30.77	0.69	1.01
G 3	17.40	11.20	0.77	35.63	0.64	0.94
Mean	15.90	10.87	0.71	31.63	0.68	1.00

Yp = grain weight/plant using pathogen and endophytic bacteria treatment (T3); Yd = grain weight/plant using pathogen (T1); PTI = pathogen tolerance index; YI = yield injury; SM = superiority measure; RP = relative performance.

With regard to the general mean of yield injury (31.63%), the G1 genotype recorded the lowest infection rate (28.49) of pathogen, followed by the G2 genotype (30.77%), while the G3 genotype was the most sensitive to infection with the pathogen (35.63%). The same results for the scale of superiority (SM) and relative performance (RP) are in harmony with the previous result of yield injury (Table 6).

The results indicate that the classification of the G1 genotype according to different indicators can be said to be distinct under all conditions compared to the other two genotypes (G2 and G3).

An environmental stress-tolerance genotype can be defined as one that gives a yield that is significantly above average under environmental stress conditions. Therefore, the results in Table 6 can separate the tested genotypes according to the grain yield / plant under pathogen infection condition (T1) and under pathogen infection condition when treated with endophyte bacteria these results are consistent with Majidi et al. (2011) and Badran and Moustafa (2014). Therefore, based on the results presented in Table 6, it is possible to divide the genotypes into three groups based on their performance, which is indicated by Fernandez (1992), that the genotypes can be classified into a number of groups based on their performance: genotype that expresses performance good under any conditions (cluster 1), genotypes that performed only well under optimal conditions (cluster 2), genotypes that gave relatively higher productivity under environmental stress conditions (cluster 3), and genotypes that performed poorly in both cases (Group 4).

## 4. Conclusion

The results indicated that there was a significant difference between chlorophyll content index of infected plants without bioagent (untreated) compared to plants bio-inoculated with endophytic bacteria. The highest mean disease incidence was on the plants without bacterial inoculum for quinoa genotype G3. A significant differences in the weight of grains/plant, was obvious when treated with pathogens (T1) compared to the treatment with pathogens and endogenous bacteria (T3), and a decreases in total aromatic amino acids appeared due to the pathogen infection, compared to the control. The genotype G3 showed the highest response in the levels of total aromatic and total neutral amino acids. The endophytic strains promoted quinoa seedling growth mainly by improving nutrient efficiency. This improvement could not be explained by their ability to induce the production of amino acids, showing that complex interactions might be associated with enhancement of quinoa seedling performance by endophytic bacteria.

The endophytic bacterial strains were able to reduce the severity of bacterial leaf spot disease by 30, 40, and 50% in quinoa genotypes G1, G2, and G3, respectively, recording significant differences compared to the negative control. The results indicated that, G1 genotype was superior in different performance indicators (pathogen tolerance index, yield injury %, superiority measure and relative performance) for grain weight/plant under pathogen infection condition when treated with endophyte bacteria.

Based on this study, these bacterial strains can be used as a biotechnology tool in quinoa seedling production and biocontrol to diminish the severity of bacterial leaf spot disease.

In fact, plant growth-promoting endophytic bacteria can offer incredible benefits to plants and can support the environmentally friendly approaches for sustainable agriculture. They can be used as tools that could be an alternative way to diminish the use of chemicals. It is obvious that, under the current climate changes and increasing world population, the sustainability of agriculture should be based on innovative environment-friendly approaches and should consider crop biodiversity and highly nutritious crops such as quinoa and other non-conventional crops.

Our study focused on the effect of PGPEB on quinoa growth and resistance to bacterial leaf spot disease. It proved that PGPEB can be used as promising bioagents to limit this disease and can be used as a biotechnology technique in quinoa seedling production in Egypt and other countries with similar climate conditions.

However, further investigation of the beneficial impacts of the tested PGPEB is required and should focus on the implications of such beneficial bacteria in plant protection management.

## Data availability statement

The datasets presented in this study can be found in online repositories. The names of the repository/repositories and accession number(s) can be found at: https://www.ncbi.nlm.nih.gov/nuccore/, OP984768, OP984769, OP984765, and OP984766.

## Author contributions

AB and NE designed the research and wrote the initial draft of this manuscript and conceived the original ideas. NE conducted the experiment and analysis. AB, NE, AH, and HM contributed to idea refinement, writing, and revising of the manuscript. HM supervised the work and revised and finalized the manuscript. All authors have read and agreed to the published version of the manuscript.

## Funding

This work has been supported by the International Center for Biosaline Agriculture (ICBA) as part of the project Developing a User-Friendly Application for Smallholder Farmers for Detection of Plant Disorders.

## Conflict of interest

The authors declare that the research was conducted in the absence of any commercial or financial relationships that could be construed as a potential conflict of interest.

## Publisher’s note

All claims expressed in this article are solely those of the authors and do not necessarily represent those of their affiliated organizations, or those of the publisher, the editors and the reviewers. Any product that may be evaluated in this article, or claim that may be made by its manufacturer, is not guaranteed or endorsed by the publisher.
